# Risk of sequelae after invasive meningococcal disease

**DOI:** 10.1186/s12879-022-07129-4

**Published:** 2022-02-11

**Authors:** Sidsel Skou Voss, Jens Nielsen, Palle Valentiner-Branth

**Affiliations:** 1 European Programme for Intervention Epidemiology Training (EPIET), European Centre for Disease Prevention and Control (ECDC), Stockholm, Sweden; 2 Infectious Disease Epidemiology and Prevention, Statens Serum Institut, Artillerivej 5, 2300 Copenhagen S, Denmark

**Keywords:** Sequelae, Invasive meningococcal disease, Epidemiology, Determinants/factors, Surveillance

## Abstract

**Background:**

Invasive meningococcal disease (IMD) is a rare but severe bacterial infection, of which a high proportion of survivors are affected by sequelae. In Denmark, IMD is a notifiable disease and data collection on sequelae information has been automated, enabling studies of sequelae due to IMD diagnosed after discharge. The aim of this study was to examine possible determinants for sequelae after IMD and to describe the distribution of sequelae by age, serogroup and clinical presentation, for all cases in Denmark from 2005–2020.

**Methods:**

Data from The National Database for Notifiable Infectious Diseases was linked to data from The Danish National Patient Register and the Civil Registration System. Logistic regression models were used to study whether age, serogroup and/or clinical presentation were associated with sequelae. A descriptive analysis of the proportion of different types of sequelae across age groups, serogroups and clinical presentations was performed.

**Results:**

In total, 25% of IMD survivors experienced one or more sequelae. We found no significant association between sequelae and age. The five most common sequelae in decreasing order of incidence were hearing loss, epilepsy, learning disabilities, headache and visual defects/loss of vision, with rates ranging from 8.2 to 2.8% of IMD survivors. The proportion of survivors with hearing loss and visual defects/loss of vision was not significantly different between clinical presentations.

**Conclusions:**

We suggest revising IMD treatment guidelines, to include routine referral to hearing and vision tests, irrespective of clinical presentation. Furthermore, it is important to increase the awareness among parents of children who have had IMD of possible future learning disabilities to make sure that necessary measures are taken in a timely manner.

**Supplementary Information:**

The online version contains supplementary material available at 10.1186/s12879-022-07129-4.

## Background

Invasive meningococcal disease (IMD) is a rare but severe bacterial infection caused by *Neisseria meningitidis*. Nasopharyngeal colonisation with *Neisseria meningitidis* is observed in 5–10% of persons, without them presenting any clinical symptoms. If the bacteria invades the body, it can cause severe illness in the form of meningitis or septicaemia (blood infection) [1]. IMD occurs worldwide with the highest incidence of the disease found in the ‘meningitis belt’ of sub-Saharan Africa. IMD can be prevented through vaccination, but a vaccine that protects against all serogroups does not yet exist. Research points towards vaccination being the driving force behind the decrease in serogroup C in many European countries, but the reason for the overall decline in IMD in recent decades, predominatly in serogroup B, is not clear [2]. In Denmark, the incidence of IMD has decreased over the past 30 years and was stable at a low level from 2014–2018, with an incidence of about 0.7 cases per 10^5^ inhabitants per year. In 2019, the incidence increased slightly to 1.0 cases per 10^5^ inhabitants, followed by a drop during the COVID-19 pandemic to 0.3 cases per 10^5^ inhabitants in 2020.

Although IMD can be treated with antibiotics, the mortality remains high (5–15%) [1, 3]. Among the survivors, 10–20% will have long-term disabilities, such as deafness, nervous system problems, brain damage or loss of limb(s) [3–5]. Additionally, psychological/psychiatric sequelae, such as anxiety, depression, attention deficit hyperactivity disorder (ADHD) [6] and emotional and behavioural difficulties have been increasingly reported in the literature in recent years [3]. Several studies found that older age and clinical presentation (septicaemia only and meningitis and septicaemia) are associated with a higher risk of a fatal course of IMD [7, 8], but to our knowledge, studies on whether certain determinants are associated with a higher risk of sequelae after IMD have not been done.

According to Danish law, IMD is notifiable to the Patent Safety Authority and the Department of Infectious Disease Epidemiology and Prevention, Statens Serum Institut (SSI). According to the guidelines for treatment of acute bacterial meningitis from the Danish Society for Infectious Diseases [9], physicians are obliged to notify the authorities of a case of meningitis within 5 days of the start of treatment. As many sequelae are diagnosed after discharge, an underreporting of sequelae from the physicians was expected. In 2014, Denmark began the automated extraction of information regarding sequelae from the Danish National Patient Register (DNPR) with the aim of reducing the amount of paperwork required of physicians and improving data quality [10]. This method enabled studies of sequelae due to IMD in which sequelae diagnosed after discharge were included, to be conducted. The primary aim of our study was to investigate whether age, serogroup and clinical presentation are determinants associated with sequelae among survivors of IMD and to describe the distribution of the different sequelae per age group, serogroup, and clinical presentation during the period 2005–2020. Another aim was to describe the methods used for automatic retrieval of information about sequelae.

## Methods

**Fig. 1:**
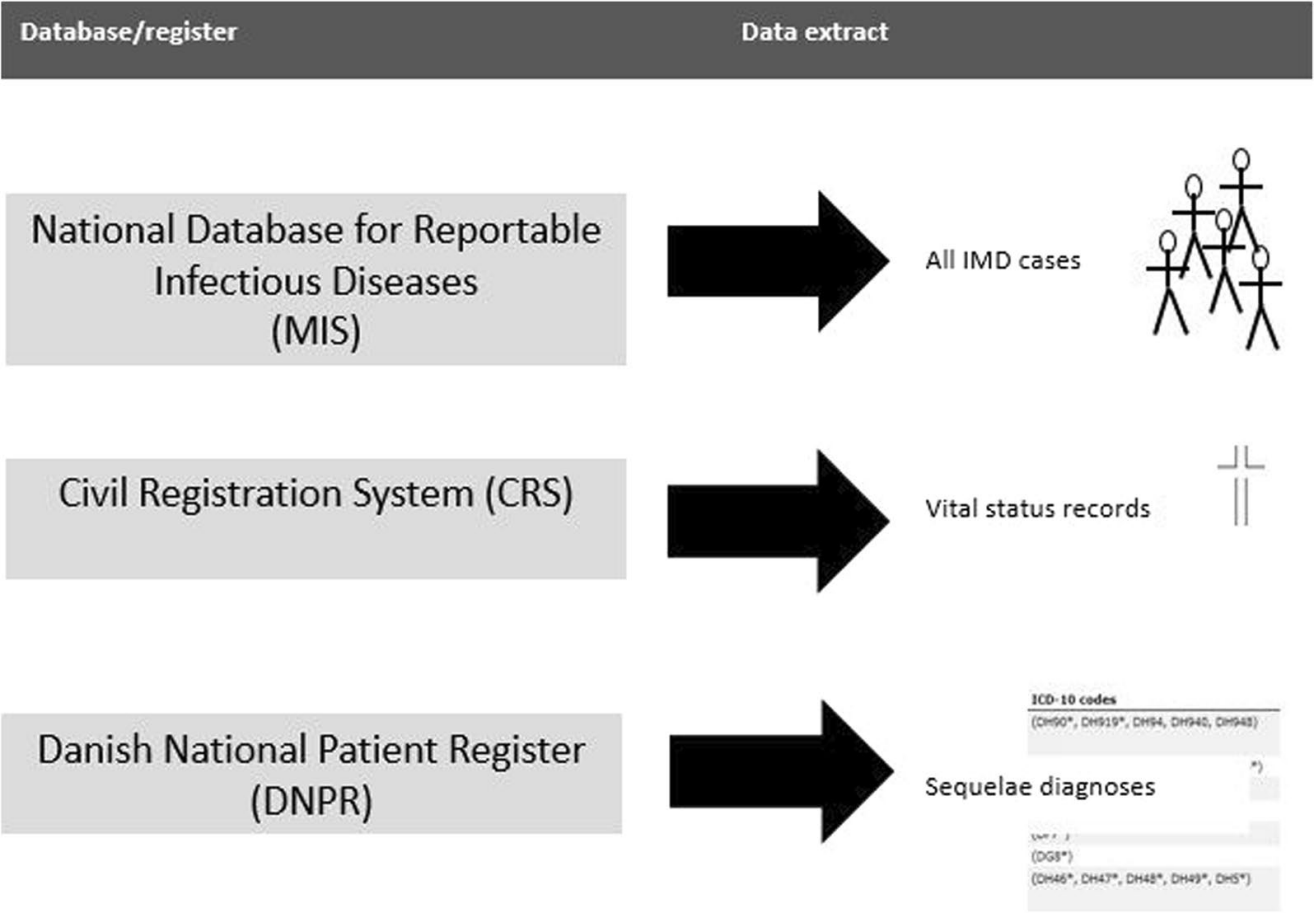
Data sources

Using registry based national data, we performed a retrospective population based study. The data was collected from the databases and registers shown in Fig. 1. In addition to the mandatory reporting of suspected and confirmed IMD cases to *The National Database for Notifiable Infectious Diseases (*MIS) at SSI, the voluntary submission of meningococcal isolates from blood, cerebrospinal fluid and/or other relevant anatomical sites by regional departments of clinical microbiology to SSI supports the ongoing surveillance. The Danish Microbiology Database (MiBa), a national database containing reports from all departments of clinical microbiology, has further supported surveillance of IMD since its establishment in 2010. The study population included survivors of IMD who were registered in MIS between 2005 and 2020. Survivors of IMD were defined as cases who were alive 30 days after microbiologically identified IMD. Vital status and date of death were extracted from the Civil Registration System (CRS). Data extracted from MIS included age, sex, date of debut, and clinical presentation (meningitis only, septicaemia only, meningitis and septicaemia, other invasive disease). Based on the current available literature [5, 11], a list of known sequelae in the form of ICD10 codes [12] was used for individual level extraction from *The Danish National Patient Register* (DNPR) (see list in Additional file 1). DNPR contains all diagnoses from admissions and outpatient contacts for all hospitals in Denmark, excluding those from general practitioners and other private specialists (e.g. dermatologists and orthopaedics). The data was linked via a unique identification number (CRS-number) given to all legal residents of Denmark.

Only sequelae registered after hospital discharge were included. Diagnoses which appeared on the defined list of sequelae but were registered both before and after the IMD-hospitalization, were excluded. Time limits were set for certain categories of diagnostic codes to define whether the diagnosis was a sequela or not (Additional file 1). For example, in adult cases, visual defects diagnosed more than 1 year after IMD were not considered to be a sequela, whereas for infants it was, due to the fact that there are limited other reasons for visual defects among infants as age-related visual impairment, cataracts, etc. do not affect this age group. The data was extracted in January 2021.

### Data analysis

Potential determinants were divided into categories. Due to small numbers, cases of serogroup A, X, Z, 29E as well as cases of unknown serogroup were pooled as “other”. The effects of the determinants on the outcome (sequelae or no sequelae) among survivors were analysed using a univariate logistic regression and a multivariate logistic regression model including all determinants and adjusted for sex. Odds ratios (OR) and 95% confidence intervals were estimated. A descriptive analysis was done for reporting frequency of sequelae among survivors of IMD cases from 2005–2020. The need for rehabilitation could reflect sequelae and the proportion of survivors who received rehabilitation was, for that reason, reported in the descriptive analysis (but not included as a sequelae in the logistic regression analyses). Statistical analyses were performed in STATA (version 14 StataCorp Texas).

## Results

Denmark had 935 IMD cases between 2005 and 2020, of which 96.5% was laboratory confirmed. Cases were seen across all ages (0–96 years, mean age 26 years). A double peak was observed for the age groups with the highest number of cases, one among children 0–5 years of age and a second peak among adolescents. Of the 935 cases, 66 died within 30 days of microbiologically identified IMD, and among the 869 survivors, 25% experienced one or more sequelae: 161 survivors were registered with only one, 45 with two sequelae, and seven were registered with three sequelae. Hearing loss was the most frequent sequelae among those registered with only one sequelae. The most frequent combinations for those registered with two sequelae were epilepsy and headache (n = 6) and epilepsy and hearing loss (n = 4).

Survivors with sequelae and those without had comparable distributions across all determinants except clinical presentation (Table 1).

**Table 1:** Characteristics of 869 survivors of invasive meningococcal disease by experiencing sequelae or not from 2005–2020

	Cases					p-value
	Total	Sequelae		No sequelae		
	n (%)	(n)	(%)	(n)	(%)	
Gender						
Male	468 (54%)	108	23	360	77	0.288
Female	401 (46%)	105	26	296	74	
Age group						
0–5	281 (32%)	62	22	219	78	0.520
6–15	142 (16%)	33	23	109	77	
16–25	179 (21%)	45	25	134	75	
26–65	163 (19%)	48	29	115	71	
66+	104 (12%)	25	24	79	76	
Serogroup^a^						
B	379 (44%)	95	25	284	75	0.078
C	232 (27%)	65	28	167	72	
W	71 (8%)	8	11	63	89	
Y	54 (6%)	13	24	41	76	
Other	131 (15%)	31	24	100	76	
Clinical presentation						
Meningitis only	315 (36%)	81	26	234	74	0.018
Meningitis and septicaemia	245 (28%)	74	30	171	70	
Septicaemia only	285 (33%)	54	19	231	81	
Other invasive disease	24 (3%)	4	17	20	83	

^a^Two cases had unknown serogroup

The proportion of cases with and without sequelae was also comparable across year of infection (Additional file 2).

The univariate analysis among survivors indicated that age and serogroup were not associated with sequelae (p = 0.523, p = 0.096), while clinical presentation was (p = 0.019). In the multivariate logistic regression, the odds of sequelae seemed to increase with age, but the trend was not significant (p = 0.21). No statistically significant association between sequelae and clinical presentation or serogroup was observed (Table 2). A negative association for cases with serogroup W compared to cases with serogroup B was found (OR 0.38; 95%-CI [0.17;0.87], p = 0.022).

**Table 2:** Univariate and multivariate logistic regressions, determinants possibly associated with one or more sequelae after invasive meningococcal disease

	Univariate			Multivariate		
	OR	95% CI	p	OR	95% CI	p
Age group			0.523			0.107
0–5	Ref			Ref		
6–15	1.07	(0.66–1.73)	0.784	1.05	(0.64–1.72)	0.851
16–25	1.19	(0.76–1.84)	0.447	1.26	(0.8–1.99)	0.310
26–65	1.47	(0.95–2.29)	0.083	1.80	(1.13–2.85)	0.013
66+	1.12	(0.66–1.9)	0.681	1.66	(0.91–3.03)	0.102
Serogroup			0.096			0.146
B	Ref			Ref		
C	1.16	(0.8–1.68)	0.421	1.14	(0.78–1.67)	0.487
W	0.38	(0.18–0.82)	0.014	0.38	(0.17–0.87)	0.022
Y	0.95	(0.49–1.84)	0.875	0.93	(0.46–1.89)	0.843
Other	0.93	(0.58–1.48)	0.748	0.97	(0.6–1.56)	0.903
Clinical presentation			0.019			0.023
Meningitis only	Ref			Ref		
Meningitis and septicaemia	1.25	(0.86–1.81)	0.239	1.33	(0.91–1.95)	0.143
Septicaemia only	0.68	(0.46–1)	0.048	0.68	(0.45–1.04)	0.074
Other invasive disease	0.58	(0.19–1.74)	0.330	0.65	(0.21–2.01)	0.459

Sequelae experienced among the 869 survivors of IMD during the period 2005–2020 are shown in Table 3. The numbers shown for each category of the three variables: age group, serogroup and clinical presentation, are the proportion of the specific sequelae in each of the categories. The number of cases with the specific sequelae are shown in Additional file 3. The most frequently registered sequelae was hearing loss (8.2%), equivalent to 35% of all cases with sequelae. The proportion varied from 6.8 to 10.6% between age groups, but was most frequently observed in cases older than 65 years and most rarely among children 0–5 years. The second most frequent sequelae was epilepsy, which was registered for 47 cases (5.4%). Occurrence of epilepsy ranged between 3.8–8.0% in the different age groups and was most frequently observed in the age group 26–65 years. The difference in distribution between age groups, serogroups and clinical presentations was not statistically significant for hearing loss nor epilepsy. The third most frequent sequelae registered was learning disabilities (3.6%), for which a significant difference between age groups was seen, with the highest proportion being among cases under 16 years. The distribution between serogroups and clinical presentations was not significantly different. The fourth most frequent sequelae was headache, which was registered for 30 cases (3.5%). The proportion was significantly different between age groups, varying from 1.4 to 6.7%, with the highest proportion seen in the age group 16–25 years and the lowest among those 0–5 years and 66+ (1.4% and 1.9%). The difference in distribution between serogroups and clinical presentation was not statistically significant. Twenty-four cases (2.8%) had visual defects or loss of vision, the fifth most frequently registered sequelae. It was most commonly observed among children 0–5 years (4.3%) and rarely among patients older than 65 years (1%). For visual defects/loss of vision, the difference in distribution between age groups, serogroups and clinical presentations was not statistically significant. Furthermore, as seen in Table 3, 2.9% (25 cases) received rehabilitation after having IMD.

**Table 3:** Proportion of different types of sequelae among the 869 IMD survivors by factor from 2005–2020

	Death within 30 days, n (%)	Total number of survivors	Hearing loss	Epilepsy	Learning disabilities*	Headache**	Visual defects and loss of vision	Abnormal involuntary movements	Other—the nervous and movement system	Arthritis***
Proportion of IMD survivors with sequelae (%)										
Age group (years)										
0–5	11 (4%)	281	6.8	3.9	5.3	1.4	4.3	1.1	0.4	1.4
6–15	2 (1%)	142	9.2	6.3	5.6	2.8	2.8	0.7	0.7	0.7
16–25	14 (8%)	179	8.4	5.6	2.2	6.7	2.2	1.7	1.1	0.6
26–65	10 (6%)	163	7.4	8	2.5	4.9	1.8	3.1	3.7	3.1
66+	29 (28%)	104	10.6	3.8		1.9	1	1	1.9	
Serogroup^										
B	26 (7%)	379	8.7	5.8	4.2	4	3.4	1.1	1.3	0.8
C	20 (9%)	232	10.8	4.3	3.9	2.6	3	1.7	1.3	1.7
W	10 (14%)	71	4.2	2.8		1.4	2.8	1.4		2.8
Y	6 (11%)	54	3.7	5.6		5.6		1.9	1.9	1.9
Other	4 (3%)	131	6.1	6.9	4.6	3.8	1.5	2.3	2.3	0.8
Clinical presentation										
Meningitis only	11 (3%)	315	9.8	6.7	2.2	4.8	2.2	1.3	2.2	0.6
Meningitis and septicaemia	27 (11%)	245	9	6.1	6.5	2	4.1	2	1.6	2
Septicaemia only	26 (9%)	285	5.6	3.9	2.8	3.5	2.1	1.4	0.4	0.7
Other invasive disease	2 (8%)	24	8.3				4.2			8.3
Total	66 (8%)	869	8.2	5.4	3.6	3.5	2.8	1.5	1.4	1.3

	Vascular disorders of the brain	Sequelae others	Mental retardation	Embolism, thrombosis, amputation, gangrene and skin necrosis	(CNS—abnormal US)	Hydrocephalus	Palsy****	Walking difficulties and mobility disorders	Rehabilitation*****
Proportion of IMD survivors with sequelae (%)									
Age group (years)									
0–5		0.7	0.7		0.4				0.7
6–15		1.4	0.7						0.7
16–25	1.7	1.1	1.1	1.1		0.6			2.8
26–65	1.2	0.6	0.6	0.6	0.6				4.9
66+	2.9	1		1	1	1	1.9	1	8.7
Serogroup^									
B	0.8	1.1	0.8	0.3	0.3		0.3		3.7
C	1.7	1.3	1.3	0.9		0.9	0.4	0.4	2.2
W		1.4							4.2
Y	1.9			1.9	1.9				1.9
Other					0.8				1.5
Clinical presentation									
Meningitis only	1.6	0.6	0.3			0.3	0.6		3.2
Meningitis and septicaemia	1.2	2	1.2	0.4	0.4	0.4			1.6
Septicaemia only		0.4	0.7	1.1	0.7			0.4	3.5
Other invasive disease									4.2
Total	0.9	0.9	0.7	0.5	(0.3)	0.2	0.2	0.1	2.9

^Two cases had unknown serogroup

^*^Significant difference in distribution between age groups (p = 0.011)

^**^Significant difference in distribution between age groups (p = 0.036)

^***^Significant difference in distribution between clinical presentation (p = 0.019)

^****^Significant difference in distribution between age groups (p = 0.005)

^*****^Significant difference in distribution between age groups (p < 0.000)

## Discussion

This study examined the association between the determinants age, serogroup and clinical presentation and experience of sequelae after invasive meningococcal disease. Among the 869 survivors, 213 (25%) developed sequelae. The odds of experiencing/developing sequelae increased for all age groups, except for 66+ years, when compared to the reference group (0–5 years). However, there was no statistical support for trend. The proportion of IMD cases with sequelae in this study is overall similar to a German study from 2020, which concluded that severe complications and sequelae were associated with extensive costs and increased usage of healthcare resources in Germany due to IMD-related hospitalization, especially in the 1st year after IMD diagnosis [13]. Orthopaedic sequelae/amputations and skin necrosis/scarring are commonly reported sequelae in IMD survivors [3, 13, 14]. We found a surprisingly low proportion of cases with embolism, thrombosis, amputation, gangrene and skin necrosis (0.5%). These severe sequelae appear during the course of the acute illness, and registration in DNPR are therefore expected. Unlikely, if orthopaedic sequelae/skin scarring by mistake are not registered by the hospital, and the patient receives treatment by private orthopedic surgeons, dermatologists or plastic surgeons later on, data would not be included in the study. The reason behind the low proportion found in our study is unknown, but early diagnosis and early initiation of treatment could play a role. Howitz et al. [7] found estimates of association between age group and fatal outcome very similar to the estimates we found between age group and sequelae. Additionally, Howitz et al. found clinical presentation to be associated with fatal outcomes.

For the 0–5 years age group, the three sequelae with highest frequency were hearing loss (6.8%), learning disabilities (5.3%) and visual defects/loss (4.3%). Hearing loss was the most frequent sequelae in all age groups and was observed in 5.6–9.8% of cases across clinical presentation. Altogether, 35% of all cases with sequelae had hearing loss. Similar results were found by Edmond et al. [5], where 7.8% of children < 16 years experienced hearing loss, and by Stein-Zamir et al. [15], where 7% of the children had hearing impairment, of which half of the children had severe hearing loss, 1.7% needed hearing aids, and 0.9% had a need for cochlear implant. The treatment guidelines for acute bacterial meningitis from the Danish Society for Infectious Diseases [9] include referral to an outpatient hearing check. According to the results of our study, the referral should also be made for IMD cases with a clinical presentation other than meningitis. Similarly, visual defects and/or loss of vision should be checked for routinely, especially among children for whom the occurrence was the highest, as the ability to detect it by themselves is small.

For children attending primary and lower secondary school (age 6 to 16 years), the frequency of learning disabilities among IMD survivors was 5.4%. Screening for learning disabilities is more complex than conducting hearing and vision tests and learning disabilities are likely to go undetected until later in life in very young cases. For that reason, our results might underestimate the occurrence of learning disabilities after IMD. Ensuring that parents are aware of the risk of learning disabilities might increase the likelihood of detection, providing the opportunity to give the child the help he or she needs to cope and/or compensate. However, the rather low proportion of learning disabilities occurring in children as a result of IMD should be mentioned to avoid unnecessary parental concern.

The surveillance system for IMD in Denmark works well and the total number of IMD cases is assumed to be accurate, as the mandatory notification system has been supported both by MiBa and the voluntary submission of meningococcal isolates from regional departments of clinical microbiology since 2010. For quality control of the automated method for obtaining data on sequelae, The Department of Infectious Disease Epidemiology and Prevention, SSI performed a retrospective non-inferiority cohort study on data from 2004–2013 (unpublished data). The study showed that the automated retrieval of data identified more cases with sequelae compared to data from the manual reporting of sequelae from treating physicians, but the number of sequelae per case was similar.

In our study, a higher number of IMD sequelae was seen compared with surveillance data from before the automated use of data from registries was implemented (data not shown). We believe these results reflect a credible assessment of the type and frequency of sequelae because we captured sequelae diagnosed after discharge using an automated extraction method of sequelae diagnoses from the Danish National Patient Registry (DNPR). In a systematic review by Olbrich et al. from 2018, similar or even higher proportions of IMD sequelae were found [3]. Despite the higher proportions of sequelae, we assume that there may be an underestimation of some sequelae, e.g. learning disabilities, due to short follow up times for cases during the most recent years, varying completeness and validity of the DNPR and the fact that the presence of learning disabilities is not always immediately apparent in infant IMD cases [16]. The automated data extraction method saves time for the treating physicians, provides high quality data, and enables the comparison of data between different years, as the same criteria are used. Furthermore, the method can be expanded if additional diagnostic codes need to be monitored, e.g. the psychiatric/psychological sequelae.

Howitz et al. examined selected neurological sequelae and found an association between a specific serogroup B phenotype (B:15:P1.7,16) and increased risk of perceptive hearing loss [7]. At SSI, whole genome sequencing is performed with all submitted meningococcal isolates from patients with IMD. Thus, possible associations between specific genotypes and sequelae may be studied in the future.

A limitations of our study includes the possibility that some diagnostic codes (sequelae) are missing, though the list of sequelae was based on findings from current scientific literature. Studies have shown that admission of children for meningococcal disease is associated with an increase in, and high levels of, psychiatric and posttraumatic stress disorder symptoms among children and parents, both in the short and long term [16, 17]. A survey in England showed that 11% of children aged 3–16 years old who had been admitted due to meningococcal disease had an increased risk of developing posttraumatic stress disorder (PTSD) within 1 year after hospitalization [16]. Another study from England found no significant difference in the prevalence of PTSD between children with serogroup B IMD and controls, but found that about one-tenth of children surviving serogroup B meningococcal disease had major disabling deficits and more than a third had one or more deficits in physical, cognitive and psychological function [18]. In the present register based study we did not include psychological sequelae, as many factors can possibly influence the development of these conditions and a questionnaire based survey would be needed to take such factors into account. Further investigation of psychiatric, as well as physical sequelae, after IMD is needed and interactions between the different sequelae would be interesting to study in this context. Above all, the focus on screening for psychological disorders and cognitive deficits, in addition to outpatient hearing checks in hospitals is of paramount importance. Additionally, the time limits we used to define whether diagnostic codes were sequelae or not were defined from a clinical perspective and could have been a limitation. Many sequelae were only included if diagnosed within 1 year of having IMD. These time limits partly explain that the proportion between cases with sequelae and cases without sequelae was comparable over the year of infection. Another limitation is the risk of underestimation as the number of sequelae in the DNPR only contains diagnosis codes given in hospitals. The general practitioners probably manage and treat some or many patients with mild sequelae (e.g. headache or mild cognitive problems). Lastly, sequelae identified through diagnostic codes cannot definitely be attributed to IMD, and the lack of a matched comparison group could be a limitation. Though, we are quite confident using this method, as we prior to this study compared data regarding sequelae registered by the treating physician in the notification form with data extracted from DNPR over a 10-year period. The study found the same number of sequelae pr. case, but a higher number of cases with sequelae, especially hearing loss (often diagnosed after discharge and therefore not registered by the treating physician). This unpublished study concluded that use of DNPR data for surveillance was not inferior to data collected via notification forms.

## Conclusions

We suggest that routine referral to hearing tests should be included in guidelines for treating IMD, regardless of clinical presentation. Similarly, visual defects or loss of vision should be routinely checked for, especially among children younger than 16 years. Making parents of children who have had IMD aware of the possibility of future learning disabilities is important to ensure that necessary measures can be taken, even if it may cause unnecessary parental concern. Automated extraction of data on experienced sequelae is a useful method that saves time for treating physicians and generates high-quality surveillance data.

## Supplementary Information

**Additional file 1.** List of sequelae after IMD (ICD10 codes) including defined criteria.

**Additional file 2. **Number and proportion of survivors of invasive meningococcal disease experiencing sequelae, by year in 2005–2020.

**Additional file 3. **Number of survivors with sequelae after invasive meningococcal disease by factor, 2005–2020.

## Data Availability

The datasets generated and analysed in the current study are not publicly available due to Danish law as they contain information that could compromise research participant privacy, but are available in aggregated form from the corresponding author on reasonable request.

## Abbreviations

CRS: Civil Registration System
DNPR: Danish National Patient Register
IMD: Invasive meningococcal disease
MIS: National Database for Reportable Infectious Diseases
OR: Odds ratio
SSI: Statens Serum Institut
