# Supplementary material for: Risk of sequelae after invasive meningococcal disease
Source: BMC Infect Dis. 2022 Feb 11;22:148. doi: 10.1186/s12879-022-07129-4 (PMC8831877; doi:10.1186/s12879-022-07129-4)
Supplement: Supplementary file 1 — Additional file 1. List of sequelae after IMD (ICD10 codes) including defined criteria. [file 12879_2022_7129_MOESM1_ESM.docx]

**Additional file 1**

Title: List of sequelae after IMD (ICD10 codes) including defined criteria

| **ICD-10 codes** | |  |  |  | **Category** |  | **Criteria** |  |  |  |
| --- | --- | --- | --- | --- | --- | --- | --- | --- | --- | --- |
| DH81*, DH90*, DH919*, DH94, DH940, DH948 | | | | | Hearing loss | | Adults: one year after discharge Children: (< 18 years) no time limit. | | | |
|  |  |  |  |  |  |  |  |  |  |  |
| DG40*, DG41*, DG43*, DG44*, DG45* | | | |  | Epilepsy |  |  |  |  |  |
| DR25* |  |  |  |  | Abnormal involuntary movements | |  |  |  |  |
| DR26* |  |  |  |  | Walking difficulties and mobility disorders | |  |  |  |  |
| DF7* |  |  |  |  | Mental retardation | |  |  |  |  |
| DG8* |  |  |  |  | Palsy |  |  |  |  |  |
| DH46*, DH47*, DH48*, DH49*, DH5* | | | |  | Visual impairment | | Adults: one year after discharge Children: (< 18 years) no time limit. | | | |
|  |  |  |  |  |  |  |  |  |  |  |
| DG91*, DG94* | |  |  |  | Hydrocephalus | |  |  |  |  |
| DG05* |  |  |  |  | Inflammation of the CNS other than meningitis | |  |  |  |  |
| DR51* |  |  |  |  | Headache |  |  |  |  |  |
| DR940* DR949* | |  |  |  | Abnormal finding on functional examination CNS | | One year after discharge | | |  |
| DR941* |  |  |  |  | Abnormal findings in functional examination   - the peripheral nervous system and sensory organs | | One year after discharge | | |  |
|  |  |  |  |  |  |  |  |  |  |  |
| DR948* |  |  |  |  | Abnormal finding on functional examination - other organ | | One year after discharge | | |  |
| DR298* |  |  |  |  | Other or unspecified or abnormal findings in the CNS | | One year after discharge | | |  |
| DR944*, DB948*, DB949* | | |  |  | Other sequelae | | One year after discharge | | |  |
| DM028*, DM029*, DM03* | | |  |  | Arthritis |  | One year after discharge | | |  |
| DI74*, KNHQ*, KNCQ*, DR02*, BNPA02 | | |  |  | Embolism, thrombosis, amputation, gangrene and skin necrosis | | One year after discharge | | |  |
| DZ55*, DZ56*, DZ57*, DZ58*, DZ58*, DZ59*,DZ6* | | | | | Learning disabilities | |  |  |  |  |
| DI6* |  |  |  |  | Cerebrovascular disease | | One month after discharge | | |  |
| BRAA* |  |  |  |  | Activity training of cognitive and intellectual functions | |  |  |  |  |
| DZ508 |  |  |  |  | Rehabilitation | | Six months after discharge | | |  |
