# Supplementary material for: Risk of sequelae after invasive meningococcal disease
Source: BMC Infect Dis. 2022 Feb 11;22:148. doi: 10.1186/s12879-022-07129-4 (PMC8831877; doi:10.1186/s12879-022-07129-4)
Supplement: Supplementary file 2 — Additional file 2. Number and proportion of survivors of invasive meningococcal disease experiencing sequelae, by year in 2005–2020. [file 12879_2022_7129_MOESM2_ESM.docx]

**Additional file 2**

Title: Number and proportion of survivors of invasive meningococcal disease experiencing sequelae, by year in 2005-2020
Description: Part of the descriptive analysis showing a comparable distribution of cases experiencing sequelae after invasive meningococcal disease across years.

|  |  | Cases | Cases | | p-value |
| --- | --- | --- | --- | --- | --- |
|  |  | total | with sequelae | |  |
|  |  | n (%) | (n) | (%) |  |
| Year | 2005 | 86 (10%) | 26 | 30% | 0.894 |
|  | 2006 | 73 (8%) | 17 | 23% |  |
|  | 2007 | 71 (8%) | 10 | 14% |  |
|  | 2008 | 63 (7%) | 15 | 24% |  |
|  | 2009 | 69 (8%) | 14 | 20% |  |
|  | 2010 | 66 (8%) | 17 | 26% |  |
|  | 2011 | 83 (10%) | 23 | 28% |  |
|  | 2012 | 55 (6%) | 13 | 24% |  |
|  | 2013 | 54 (6%) | 12 | 22% |  |
|  | 2014 | 39 (4%) | 9 | 23% |  |
|  | 2015 | 37 (4%) | 8 | 22% |  |
|  | 2016 | 35 (4%) | 10 | 29% |  |
|  | 2017 | 35 (4%) | 8 | 23% |  |
|  | 2018 | 34 (4%) | 8 | 24% |  |
|  | 2019 | 50 (6%) | 10 | 20% |  |
|  | 2020 | 19 (2%) | 6 | 32% |  |
