# Supplementary material for: Risk of sequelae after invasive meningococcal disease
Source: BMC Infect Dis. 2022 Feb 11;22:148. doi: 10.1186/s12879-022-07129-4 (PMC8831877; doi:10.1186/s12879-022-07129-4)
Supplement: Supplementary file 3 — Additional file 3. Number of survivors with sequelae after invasive meningococcal disease by factor, 2005–2020. [file 12879_2022_7129_MOESM3_ESM.docx]

**Additional file 3**

Title: Number of survivors with sequelae after invasive meningococcal disease by factor, 2005-2020

Description: The number of survivors with sequelae after invasive meningococcal disease are the numerators in the calculations of proportions of survivors experiencing sequelae in table 3.

|  | |  | |  | |  | |  | |  | |  | |  | |  | |  | |  | |  | |  | |  | |  | |  | |  | |  | |  | |  | |  | |
| --- | --- | --- | --- | --- | --- | --- | --- | --- | --- | --- | --- | --- | --- | --- | --- | --- | --- | --- | --- | --- | --- | --- | --- | --- | --- | --- | --- | --- | --- | --- | --- | --- | --- | --- | --- | --- | --- | --- | --- | --- | --- |
|  | |  | |  | |  | |  | |  | |  | |  | |  | |  | |  | |  | |  | |  | |  | |  | |  | |  | |  | |  | |  | |
|  | |  | | Death within 30 days | | Total number of survivors | | Hearing loss | | Epilepsy | | Learning disabilities | | Headache | | Visual defects and loss of vision | | Abnormal involuntary movements | | Other - the nervous and movement system | | Arthritis | | Vascular disorders of the brain | | Sequelae others | | Mental retardation | | Embolism, thrombosis, amputation, gangrene and skin necrosis | | CNS - abnormal US | | Hydrocephalus | | Palsy | | Walking difficulties and mobility disorders | | Rehabilitation | |
|  | |  | | | |  | | Number of IMD cases with sequelae (n) | | | | | | | | | | | | | | | | | | | | | | | | | | | | | | | |  | |
|  | |  |  |  |  |  | |  |  |  |  |  |  |  |  |  |  |  |  |  |  |  |  |  |  |  |  |  |  |  |  |  |  |  |  |  |  |  |  |  | |
| **Age group (years)** | | | |  | |  | |  | |  | |  | |  | |  | |  | |  | |  | |  | |  | |  | |  | |  | |  | |  | |  | |  | |
|  | | 0-5 | | 11 | | 281 | | 19 | | 11 | | 15 | | 4 | | 12 | | 3 | | 1 | | 4 | |  | | 2 | | 2 | |  | | 1 | |  | |  | |  | | 2 | |
|  | | 6-15 | | 2 | | 142 | | 13 | | 9 | | 8 | | 4 | | 4 | | 1 | | 1 | | 1 | |  | | 2 | | 1 | |  | |  | |  | |  | |  | | 1 | |
|  | | 16-25 | | 14 | | 179 | | 16 | | 10 | | 4 | | 12 | | 4 | | 3 | | 2 | | 1 | | 3 | | 2 | | 2 | | 2 | |  | | 1 | |  | |  | | 5 | |
|  | | 26-65 | | 10 | | 163 | | 12 | | 13 | | 4 | | 8 | | 3 | | 5 | | 6 | | 5 | | 2 | | 1 | | 1 | | 1 | | 1 | |  | |  | |  | | 8 | |
|  | | 66+ | | 29 | | 104 | | 11 | | 4 | |  | | 2 | | 1 | | 1 | | 2 | |  | | 3 | | 1 | |  | | 1 | | 1 | | 1 | | 2 | | 1 | | 9 | |
| **Serogroup^#^** | | | |  | |  | |  | |  | |  | |  | |  | |  | |  | |  | |  | |  | |  | |  | |  | |  | |  | |  | |  | |
|  | | B | | 26 | | 379 | | 33 | | 22 | | 16 | | 15 | | 13 | | 4 | | 5 | | 3 | | 3 | | 4 | | 3 | | 1 | | 1 | |  | | 1 | |  | | 14 | |
|  | | C | | 20 | | 232 | | 25 | | 10 | | 9 | | 6 | | 7 | | 4 | | 3 | | 4 | | 4 | | 3 | | 3 | | 2 | |  | | 2 | | 1 | | 1 | | 5 | |
|  | | W | | 10 | | 71 | | 3 | | 2 | |  | | 1 | | 2 | | 1 | |  | | 2 | |  | | 1 | |  | |  | |  | |  | |  | |  | | 3 | |
|  | | Y | | 6 | | 54 | | 2 | | 3 | |  | | 3 | |  | | 1 | | 1 | | 1 | | 1 | |  | |  | | 1 | | 1 | |  | |  | |  | | 1 | |
|  | | Other | | 4 | | 131 | | 8 | | 9 | | 6 | | 5 | | 2 | | 3 | | 3 | | 1 | |  | |  | |  | |  | | 1 | |  | |  | |  | | 2 | |
| **Clinical presentation** | | | |  | |  | |  | |  | |  | |  | |  | |  | |  | |  | |  | |  | |  | |  | |  | |  | |  | |  | |  | |
|  | | Meningitis only | | 11 | | 315 | | 31 | | 21 | | 7 | | 15 | | 7 | | 4 | | 7 | | 2 | | 5 | | 2 | | 1 | |  | |  | | 1 | | 2 | |  | | 10 | |
|  | | Meningitis & septicaemia | | 27 | | 245 | | 22 | | 15 | | 16 | | 5 | | 10 | | 5 | | 4 | | 5 | | 3 | | 5 | | 3 | | 1 | | 1 | | 1 | |  | |  | | 4 | |
|  | | Septicaemia only | | 26 | | 285 | | 16 | | 11 | | 8 | | 10 | | 6 | | 4 | | 1 | | 2 | |  | | 1 | | 2 | | 3 | | 2 | |  | |  | | 1 | | 10 | |
|  | | Other invasive disease | | 2 | | 24 | | 2 | |  | |  | |  | | 1 | |  | |  | | 2 | |  | |  | |  | |  | |  | |  | |  | |  | | 1 | |
| **Total** | |  | | 66 | | 869 | | 71 | | 47 | | 31 | | 30 | | 24 | | 13 | | 12 | | 11 | | 8 | | 8 | | 6 | | 4 | | 3 | | 2 | | 2 | | 1 | | 25 | |

# Two cases had unknown serogroup
